# Increase of myocardial infarctions over the years after heart transplantation detected by late gadolinium enhanced MRI

**DOI:** 10.1186/1532-429X-15-S1-P279

**Published:** 2013-01-30

**Authors:** Maria Fernanda Braggion Santos, Jan Simpfendörfer, Mohamed A Abdelrazek, Sebastian A Seitz, Arnt Kristen, Dirk Lossnitzer

**Affiliations:** 1 Universitatsklinikum Heidelberg, Heidelberg, Germany; 2 School of Medicine of Ribeirao Preto University of Sao Paulo, Sao Paulo, Brazil; 3 Radiology, Cairo university, Faculty of Medicine Radiology, Cairo, Egypt

## Background

Cardiac allograft vasculopathy (CAV) is one of the leading causes of death in patients who survive the first year after heart transplantation (HTX). Coronary angiography detects the presence of vasculopathy in 10% to 20% of transplant recipients at 1-year follow-up whereas CAV is diagnosed in 52% of survivors 10 years after HTX. As recently shown, non-invasive late gadolinium contrast enhanced MRI (LGE-CMR) is able to detect myocardial infarction (MI) typical patterns in patients after HTX. Since the presence of CAV over the years after HTX increases significantly, we hypothesized that MRI could detect MI consistent with the detection of CAV by coronary angiography.

## Methods

139 patients were divided into 4 groups depending on the time after HTX they were scanned (group I: 0-2 yrs, group II: 2-5 yrs, group III: 5-8 yrs and group IV: 8-10 yrs). Cine MRI with 32 channel image acquisition and vector-ECG gated short axis, two and four chamber cine slices with parallel image acquisition covering the entire left ventricle (LV) were acquired using a regular SSFP sequence on a 1.5T Whole Body MRI scanner (Achieva 1.5T, Philips Medical Systems). LGE-CMR (Gadolinium-DTPA:0.2mmol/kg, Magnevist) was performed and analyzed by two experienced blinded observers. Areas of infarct-typical LGE patterns were defined as sub-endocardial LGE patterns of various degrees of transmurality. Data were expressed as percentage and groups were compared using Chi Square or Fisher Exact Test. P-values ≤ 0.05 were considered statistically significant.

## Results

**Figure 1:**
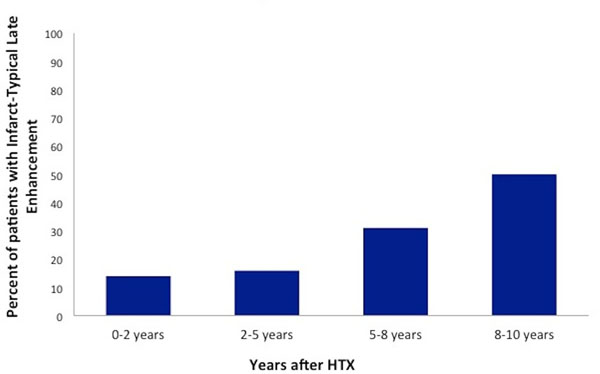
Distribution of patients with infarct-typical late enhancement over the years after heart transplantation

In group I, 12 of 87 patients (14%) showed infarct-typical LGE patterns; in group II, 16% of 26 patients; in group III, 31% of 20 patients whereas in group IV, 3 of 6 patients (50%) also had infarct-typical pattern in LGE-CMR (Figure 1). The difference among the groups did not reach statistical significance, with a trend between the groups I and IV (p= 0,051).

## Conclusions

LGE-CMR is a sensitive imaging technique to detect MI as a result of CAV since the earliest periods after HTX whereas the presence of infarct-typical patterns increases over the years. Interestingly, in our study the presence of LGE was found in 14% of the patients early and in 50% later after HTX, which appears to be similar to the invasive detection of the presence of CAV that has been previously published in the literature.

## Funding

none

